# Barriers and facilitators to health during prison reentry to Miami, FL

**DOI:** 10.1371/journal.pone.0285411

**Published:** 2023-10-30

**Authors:** Sofia Mohammad, Ahzin Bahrani, Minji Kim, Kathryn M. Nowotny

**Affiliations:** 1 University of Miami Department of Sociology and Criminology, Coral Gables, FL, United States of America; 2 University of Miami School of Medicine, Miami, FL, United States of America; Nelson Mandela University, SOUTH AFRICA

## Abstract

**Background:**

People face numerous barriers to reentry and community integration following incarceration, and these obstacles manifest themselves as barriers to economic stability, housing security, healthcare, community acceptance, and educational attainment, ultimately leading to poor health. This study aims to understand healthcare needs of reentrants post release within the unique political and service context of Miami Dade County, FL, and seeks to uncover the structural facilitators and barriers to sustaining health during reentry.

**Methods:**

We report on a subset of data from a larger reentry asset mapping project. We conducted a qualitative thematic analysis based on 12 semi-structured interviews with community stakeholders, including reentrants who were released in the past year (n = 5) and with community providers who have provided support services to returning citizens for at least one year (n = 7). Narratives were coded through an iterative process using NVivo software and were analyzed using the general inductive approach.

**Results:**

Three themes emerged from the analysis: (1) social and structural barriers and facilitators to health during reentry, (2) challenges with medical care following release, and (3) long-term impacts of receiving poor healthcare in prison. Reentrants describe the carceral environment as non-conducive to health and cite an urgent need for systemic change within correctional institutions to promote their well-being. Respondents identified substance use disorder (SUD) treatment, trauma informed therapy, and chronic disease management as the primary healthcare needs of reentrants, and cite social support, stable housing, education, and employment as the key social and structural needs upon release.

**Conclusion:**

This study identifies prevalent resource gaps in Miami Dade County during reentry. Respondents advocate for more inclusive governmental housing programs, Medicaid expansion, and more holistic reentry programs to support reentrants. Understanding the barriers and facilitators to health during reentry can inform future interventions to better support reentrants in their transition post-incarceration.

## Introduction

During 2020, approximately 1,215,800 people were incarcerated within correctional institutions in the United States, and 549,600 people were released back to the community [1]. Despite the high volume of people released each year, there is a notable lack of reentry support services to help reentrants access essential healthcare, secure housing and employment, and access essential social support services upon release [2–4]. If these essential needs remain unaddressed, reentrants are placed at a higher risk for experiencing poor physical, mental, and social health outcomes that can ultimately serve as catalysts for re-incarceration and pre-mature death [5–8].

The carceral environment in U.S. correctional institutions is exceedingly stressful and provides minimal opportunities for people to achieve and sustain their health. The majority of U.S. prisons are overcrowded, giving rise to high rates of interpersonal violence [9, 10], infectious disease transmission [6, 11–13], and mortality due to the poor quality of healthcare available in these densely populated institutions [14, 15]. Many people who are incarcerated report experiencing healthcare-induced trauma from the poor quality care they received in prisons and jails, leading to their severe fear of experiencing serious illness and death, as well as deep mistrust of healthcare providers and medical institutions as a whole [16]. These feelings of intense mistrust from adverse healthcare experiences continue into reentry, contributing to poor health outcomes post-release.

The reentry period presents a crucial opportunity for intervention, with reentry support services spanning the domains of healthcare, housing, employment, and social support proving to be especially beneficial for returning citizens as they navigate unfamiliar systems post-release, helping them achieve better health [17, 18]. Ensuring that formerly incarcerated individuals have the resources and support needed to overcome unfulfilled social determinants of health post-release can mitigate the range of inequalities that justice involved individuals face.

We examined the barriers and facilitators to health during reentry in the context of Miami-Dade County, where a sizeable proportion of Florida’s 26,420 reentrants are released each year [19]. Florida has the third largest prison population and ranks 10^th^ in imprisonment rates [20]. There are over 350,000 people under correctional supervision, with a 4:1 Black to White imprisonment ratio [21]. Miami is also the “epicenter” of the US housing crisis [22] and continues to face a high prevalence and incidence of HIV [23], with Florida being one of 12 states that has not expanded Medicaid. It is within this context that we conducted semi-structured interviews with people recently released from prison to Miami and Miami-area professionals who provide reentry support services, helping us gain insight into the process of community re-integration.

## Materials and methods

The data reported in this paper were collected as part of a larger community needs and resource assessment project. In local communities, there are often a myriad of agency programs and services designed to meet the needs of specific populations that typically must meet specific criteria often resulting in fragmented and disconnected services at the community-level [24]. Additionally, many communities lack a shared knowledge and vision of the resources necessary to support different groups. Asset mapping is a community based participatory research approach originally designed as an asset-based community development (ABCD) strategy for community building and community capacity building [24, 25]. The World Health Organization (WHO) has recognized asset-based approaches to health as critical for supporting overall population health and in reducing health disparities. Using informational telephone interviews, we identified 171 community assets and providers in Miami Dade County that were able to serve the needs of formerly incarcerated individuals during reentry. These assets were mapped using an interactive platform. We then optimized the map by conducting online surveys with a subset of the community assets. We simultaneously conducted semi-structured interviews with community stakeholders, including recent reentrants and people who provide services to people during reentry. The purpose of key informant interviews is to collect information from a wide range of people who have firsthand knowledge about the community of interest [26]. We determined that a small sample size would be sufficient since interviews will be narrowly focused and participants will have high levels of expertise [27]. We strategically identified persons for initial interviews based on local knowledge and then used a snowball sampling technique to recruit additional participants [28].

While both groups are important community stakeholders for reentry, we use specific terms to differentiate the samples for reader clarity. We use terms like “reentrants” and “formerly incarcerated” to describe the former, and terms like “professionals” to describe the latter given the inclusion criteria (see below). Professionals encompassed a range of perspectives representing different sectors, including physicians, judges, nonprofit leaders, and support group facilitators. It is important to note that these are not mutually exclusive categories but determined which interview guide [S1 and S2 Appendices] was used during the interview. For example, one participant was formerly incarcerated and provided services to people during reentry and was interviewed following the professional interview guide.

Semi-structured interviews allowed for consistent topics to be addressed across all interviews, yet the interviewer had the freedom to probe for more details that were unique to each participant’s experience. *A priori*, our research interest was in assessing the health and health related needs of people within the context of reentry to Miami Dade County from a diverse group of stakeholders. In both our participant interviews and in our analysis, the term “holistic reentry programs” is used to describe reentry programs that address all social determinants of health by providing opportunities for housing, job training/employment, healthcare access, educational training, and community involvement.

In our initial study design, we aimed to conduct 8 to 10 interviews with each group of participants (n = 16–20 total). Data collection occurred during the height of the COVID-19 pandemic (summer 2020), which made recruitment challenging, resulting in a smaller sample size than planned. However, determining sample size *a priori* in qualitative research is considered inherently problematic [29]. And despite the controversies over the use of saturation in qualitative research [30–32], we think that no new themes would be identified by conducting more interviews. Inclusion criteria for formerly incarcerated participants was that participants must (1) be an adult aged 18 years or older who was released from an adult prison to Miami Dade County within the past year, (2) speak and understand English; and (3) currently reside in Miami Dade County. Inclusion criteria for professional participants included (1) be an adult aged 18 years or older, (2) work in Miami Dade County, (3) speak and understand English, and (4) have primary job responsibilities working with or providing services to formerly incarcerated adults for at least one year (e.g., judges, parole officers, substance use counselors, reentry housing staff, etc.). Given that interviews were conducted virtually and remotely, potential participants were also assessed for their capacity to conduct the interviews through either phone call or video call. We recruited participants through referrals from community organizations and subsequent snowball sampling. Interviewers assessed the capacity of respondents to provide their informed consent and ensured that respondents understood the nature of the research, study goals, risks, and benefits before they could participate in the study. All study protocols were approved by the University Institutional Review Board at University of Miami and informed written and verbal consent was obtained from all participants included in this study.

In total, 12 participants were interviewed: 5 formerly incarcerated persons and 7 professionals. A.B. (a sociology doctoral candidate and woman of color) conducted interviews with formerly incarcerated participants and M.K. (a medical student and woman of color) conducted interviews with professionals from June 2020 through August 2020, with interviews ranging from 45 minutes to 1 hour. Each participant was interviewed at one time point, and they were not contacted for elaboration or clarification purposes. S.M. (an undergraduate pre-med student at the time of data collection and woman of color) collected additional asset mapping data that is not reported in this manuscript. All three research assistants received extensive training from the project principal investigator (K.M.N., a white woman faculty member). Formerly incarcerated respondents were compensated $30 for their time, and professionals were not compensated because providing feedback for community organizations and initiatives was inclusive of their primary job responsibilities. Study procedures were approved by the Institutional Review Board at [University] and verbal consent was obtained from each participant. We followed the consolidated criteria for reporting qualitative studies (COREQ; [33].

We transcribed interviews verbatim and used NVivo software to conduct qualitative analysis using the general inductive approach [34]. This analytic theory uses data as a mechanism for revealing the main themes that are reflected in the respondent interviews by providing a straightforward approach for deriving findings in the context of focused research questions. Three researchers from our team participated in the coding process, with one researcher coding all respondent interviews through an iterative process (S.M.), and two others reviewing the coding framework to ensure the validity of the identified codes and themes (A.B. and M.K.). As the greater themes emerged, participants’ narratives were used to explain the theme in the language that was used by participants.

## Results

### Social and structural barriers and facilitators to health during reentry

Participants identified several barriers and facilitators to healthy living in Miami upon release from prison, including (1) inclusive and affordable housing, (2) employment, (3) access to healthy food, (4) empowerment and social support, and (5) unaddressed trauma.

#### Inclusive and affordable housing

Despite stable housing being a well-established need among participants, there is an overwhelming lack of transitional housing and affordable housing for reentrants in Miami, with many being barred from housing assistance programs due to histories of drug use or criminal records. Alicia, the director of a reentry program, lamented that “[people] coming out of prison don’t meet the HUD [US Department of Housing and Urban Development] definition of homeless, so they don’t even qualify to go into a homeless shelter—even assuming that would be a healthy and productive place for them to be—which it’s not.” Sergio, a harm reduction provider, similarly rejected the “idyllic” image of “a person waiting on the outside [of a correctional institution] with a car to take [reentrants] home,” exposing the reality that the majority of reentrants “immediately have to go back to living on the street and dealing drugs to make money—whatever they have to do to eat.” He and other stakeholders postulated that “in an ideal society, you would probably have a completely integrated system where the person left prison, they already have a case manager who sets them up with jobs, [they] immediately go into some housing situation… and have a safe place to shower and get dressed for a job interview, etc.” Tanya, who was released from prison less than one year ago, participated in a reentry program upon release from prison and stated that “coming out of prison and having a job and a safe place to live… obviously took away so much stress and worry—especially at a time when it’s not so easy to get employment.” Given the relative scarcity of reentry programs of this nature, reentrants often find themselves in situations where they lack stable housing post-release, thus heightening their vulnerability.

Reentrants with substance use disorders face further barriers to accessing housing given drug related stigma. Sergio related that “you cannot go into a shelter and bring your drugs with you. That means that if you’re going to be in that shelter for 10 hours, you’re going to go through withdrawal symptoms in a couple of hours and can’t do anything about it.” Many reentrants who have engaged in an inpatient rehabilitation facility are in need of rehabilitative and supportive housing environments like halfway houses or three-quarter houses, which are housing facilities that offer greater structure and support for those seeking to maintain their sobriety before they pursue more permanent housing arrangements. Despite halfway and three-quarter houses being viable options for many houseless reentrants who seek to “maintain a sober living environment with peer support,” Miranda, a judicial member with experience presiding over drug court, explained that a notable barrier exists in that many of these houses are not inclusive to people taking medication assisted treatments like methadone and suboxone. These barriers prevent reentrants undergoing substance use disorder treatment from accessing affordable and available housing.

#### Employment

Participants maintained that employment and job training opportunities are critical components to successful reentry. However, as Miranda noted, “finding a job with a felony on your record and a former prison sentence is very, very difficult.” With scarce opportunities for job training in prison, George, who runs a reentry program focused on job training, described how some people “just need help with resume development and can go get a job,” while others “need a gentle nudge, and need job referrals and job training [because] they just have no skills.” Alex, who leads a drug recovery support program, recounted that after several years of leading support meetings in prisons, he has learned that lack of employment during the reentry period undoubtedly fuels recidivism, stating that “[reentrants] are in this ‘catch22’ cycle where they can’t get a job, they’re back in the street, they’re stealing, they’re robbing, they’re doing drugs again, and boom, they’re back in [prison] and the [offenses] get worse and worse and they end up in a downward spiral.” Jamal, who works at a reentry program and is himself formerly incarcerated, explained that the scarcity of job training opportunities coupled with poor educational attainment renders many reentrants “not [even] knowing how to fill out an application,” and asserted that upon release, reentrants need immediate linkage to job training and employment before they fall into the same behavioral patterns that initially got them arrested.

#### Access to healthy food

Access to healthy food is a problem for many people. Tanya, a Latina woman in her 30s who was recently released from prison, said that she gained around 80 pounds during her time in prison and explained how the poor prison diet hurt her physical health over the duration of her sentence: “It was unhealthy. It was bad… It’s like, you don’t even know what you’re eating like 90% of the time. So, you don’t ever want to eat that food.” Now that she is adjusting to life outside of prison, Tanya is trying to maintain a healthy diet, but she relies on food stamps and non-profit food pantry services to access food. Similarly, Aaliyah was released from prison within the past year and stated that she spent most of her life eating healthy foods and exercising through aerobics, running, and even hula hooping. She was shocked when she was told that her prison blood work indicated that she was now at risk for having a heart attack due to a spike in her cholesterol level. Aaliyah reported that she was having a difficult time getting her health “back on track” after release from prison.

#### Empowerment and social support

Participants described the positive health impacts of having a reliable support system and expressed the importance of having social support during reentry from family, friends, significant others, and support groups. For example, Richard, who was incarcerated for 8 years, noted that connections helped him “get his needs met a lot quicker than the average person that was coming out and didn’t have anyone.” Kenneth was released from prison two years ago but was on house arrest for one year. He described the reentry period as a vulnerable and lonely time due to internalized shame and stigma that causes him to feel like “I’m this criminal, and people know I’m coming, so they don’t want to be around.” Kenneth also reported feeling unable to open up to people in his peer group out of fear that he would be seen as “just another hustle for money, or food.” Participants explained that feelings of shame and guilt prevented them from reaching out to their families for fear that they would become a burden. Miranda, the judge, explained that many reentrants have “fractured relationships with their families, significant others, parents, and children” leading to low levels of familial support for many during the reentry period. These factors cause many reentrants to miss out on the positive health benefits of pro-social behaviors during reentry, ultimately heightening their isolation from their friends and families during this vulnerable transition period.

However, it was reported that education serves as a powerful mechanism for empowerment. Richard shared that “the whole aspect of just getting locked up [is seen] in some circles as a badge of honor,” stating that many people in his community felt that “jail, [is] like, it’s kind of like a blessing… it’s supposed to like give you time to like see where you messed up at and come back out even stronger, you know what I mean.” However, he elaborated that his perspective has changed significantly ever since he has had access to educational resources about the history of mass incarceration in the US. He shared:

“[When] I started reading, and you know, I guess I woke up, you know, then it was like wait a minute man, I’m a fucking slave just like everybody else like, this shit is slavery. So, fuck! Like this shit is slavery. I don’t know what else words to um describe it like… this is not a badge of honor at all. This is just… It’s like a setup… like you’re just kind of being funneled into it. [One day] you know, you kind of wake up and you realize, ‘Hey, what the hell am I doing, what did I get myself into.’ This was part of the trap, you know when you actually see how set up uh everything is, you know, um designed specifically for you to get jammed up and, and get caught in that trap. You kind of realize like, man, I have been hoodwinked, bamboozled, you know what I mean.”

The realization that the carceral system, as it is currently, is a “set up” designed specifically to entrap vulnerable populations into a cycle of poverty and incarceration has empowered participants like Richard to engage in rehabilitative practices to avoid having their freedom compromised by an unjust system. Richard went on to note the need for social support:

“If that person isn’t strong enough mentally, they’ll just repeat some crime and go back and I, and I know people that have done that, like that, that they couldn’t function properly on the outside because they were so accustomed to what was happening in there and they liked it. And they couldn’t function so they just, so they just did a crime so they could go back to prison for longer than what they what they were in there for *because they uh didn’t have a family*, *felt like no one cared about them*, *no one you know um wanted them*, *like they didn’t feel wanted*, so it’s just like, what am I, what am I here for, like? I’m not going to kill myself, but I don’t want to be here.” [emphasis added]

In addition to the practical benefits of engaging in job training and employment opportunities, Shayla described that the support she received from Alicia’s reentry program instilled a strong sense of confidence in her, and she no longer feels “embarrassed” of her background as a formerly incarcerated person. As a paid employee at her reentry program, Shayla regularly interacts with community members, stating that she routinely “[talks] to people about the [reentry] program and what [she’s] been through, and [she’s] not embarrassed because it’s… gonna end up making [her] be better.” She shared that being open about her life experiences with incarceration helps her educate people who would not otherwise know about the challenges incarcerated people experience, and she feels empowered about the lifestyle changes she has accomplished post-release. This sense of confidence and empowerment from her job training has spread to other areas of her life, and Shayla shared that while she would have normally hidden the fact that she was recently released from prison from her doctors, peers, and employers, she no longer feels ashamed to be transparent about her past.

#### Unaddressed trauma

Behavioral and mental health issues were a shared concern among both reentrants and professionals, with most participants citing an unmet need for trauma informed therapy in Miami. Miranda observed that “most cases in criminal court [are rooted in] untreated addiction, mental health, violence, child neglect, etc., and there’s very little in the law to inform the court about how to help people.” Miranda believes that recidivism during reentry is fueled by unrealistic community supervision conditions such as “don’t use illicit drugs”, “stay employed,” “don’t go around friends who are using drugs,” and “don’t change your residence” given that “it is kind of impossible for [reentrants] to satisfy these conditions without appropriate levels of treatment and rehabilitation.” She asserted that fulfilling these requirements is especially tedious for reentrants with unresolved trauma.

Alicia similarly speculated that “if people did not face intense childhood trauma or if sexual trauma was not a factor, they definitely suffered trauma in prison a hundred percent without exception, and that also has to be addressed.” She went on to say “[even] if we found each and every person awesome housing that’s safe and affordable, and we found them employment, I believe that they would still have trouble functioning. I think it is the psychosocial [issues] that are at the root, and once that’s addressed, it would enable people to be receptive to workforce training, job placement, and all those things.” Participants advocated for providing reentrants with therapy that uses a trauma-informed approach to address deeply rooted psychosocial issues. This approach seeks to gain an awareness of the widespread impact of trauma on life experience and relationships, and recognizes trauma’s role in the outlook, emotions, and behavior of the person with a history of trauma. Alicia observed that for the people she works with trauma informed care upon release “has been the most crucial service… because people were able to address their trauma with trained professionals for the first time in their lives.” However, she noted that significant challenges exist because “when people get out, they’re faced with day-to-day challenges with finding work that pays a living wage, and housing.”

### Challenges with medical care following release

There were three major challenges to medical care for people returning from prison to Miami identified by participants: (1) access to medical records, (2) lack of medical coverage, and (3) continuity of care.

### Access to medical records

All formerly incarcerated persons had some type of interaction with the healthcare system in prison; however, they were unable to access their records post-release. Though they have a legal right to their medical records, the process to acquire the paperwork was reportedly extremely bureaucratic and tedious to the point that returning citizens feel they are ultimately unable to obtain the proper documentation. Alicia lamented that “the bureaucracy can be frustrating to navigate, and people just give up, quite honestly.” For example, Tanya stated:

“I couldn’t get my records. That was another thing—the um DOC would not give me my medical records… They wouldn’t give me anything. And um it’s pretty much pointless trying to get those… They’ll say I got my medical records. What I got was my exit bloodwork. It was a sheet of paper, one sheet of paper. But as far as what happened to me within five years there medically, I have, you know, I have mammograms and all kinds of things that they had scheduled for me, but I have none of the results. They don’t give me that.”

The lack of access to prior medical records forces many reentrants to begin rebuilding their health records from scratch. Aaliyah described having “to go through a community health agency’s intake process,” stating that it took three separate appointments to complete the intake process before they were able to treat her known chronic health conditions diagnosed in prison.

#### Lack of medical coverage

Formerly incarcerated participants stated that they have been uninsured since they were released, and expressed that a lack of affordable, inclusive health insurance options serves as a potent barrier towards accessing essential healthcare services during reentry. Miranda noted that “[since Florida] is not a Medicaid expansion state, [many reentrants] have no health insurance,” stating that the exclusion of justice involved populations from governmental support services prevent reentrants from achieving health during reentry. Richard similarly lamented that the current healthcare system forces socioeconomically disadvantaged people like himself to self-medicate to cope with chronic conditions, ultimately straining them physically, mentally, and financially. Miranda and other professionals supported the notion that unmet needs of reentrants can largely be attributed to “services [not existing] or [that reentrants don’t] know how to gain access [to them].” Having worked with hundreds of people experiencing reentry, Alicia described that “it is not easy to access services on your own… and 70% of those that need access to the public health system don’t get it, but we [have] a fairly, relatively speaking, resource rich community.”

A lack of affordable dental healthcare was specifically noted as a reentry need, with participants citing that the extractions and poor-quality fillings received in prison impaired nutrition and self-esteem and caused chronic pain. For instance, Alicia stated that “[poor dental health] is a function of previous addiction” and “[a lot of formerly incarcerated people] have significant dental issues. A lot of it is medical, but a lot is cosmetic, which impacts their ability to get work. It also impacts their self-esteem.” Shayla, a reentrant, supported this notion as she shared her experience of getting her cavity treated while in prison. She explained that “in DOC they don’t like to do fillings. They like to pull your teeth. So, I had a cavity in a canine that they just pulled—so now I’m having problems with other teeth and trying to get something into place of that tooth. So that’s really messing with my self-esteem.” Since she has been released, Shayla has been having a difficult time finding a solution. Working at a low-paying job with no means of transportation, she must schedule time off, order a rideshare, and find a sliding scale medical center that provides dental work, which she feels is an impossible task.

#### Continuity of care

Continuity of care post-release is major challenge because there are no integrated systems in Miami to help reentrants continue their medications or treatment plans when they return to the community. Through Sergio’s experiences with providing harm reduction services, he described that for HIV positive individuals, continuing their medications post-release is essential because “if you’re on HIV medication and your viral load is suppressed, you don’t want to mess that up by not taking your medications because [if you stop,] your viral load is going to go up rapidly, ultimately risking resistance to an otherwise effective medication.”

Sergio continues, stating that the lack of continuity of care post-release can prove to be detrimental for reentrants with histories of injection drug use because they are extremely vulnerable to drug-related overdoses in the days following release from prison. He cautions that the transition from “enforced abstinence—or close to it from any drug use—to the freedom to use again” has led to fatal overdoses for many of their participants following release. For this reason, he believes an integrated system can support his program in ensuring that reentrants “do not immediately fall back into some level of harm that will be fatal…that they will not overdose the day after they get out of jail.” Sergio states that since the harm reduction program he works with provides anonymous services and is not a government agency, they have limited information about when their program participants become incarcerated, or when they are released. Currently, his program facilitates continuity of care by informally asking program participants if they’ve seen a particular person recently to probe for information about whether or not a participant has been released back to the community. While this system is not ideal, it is currently the only mechanism in place in Miami to connect reentrants with histories of injection drug use to rehabilitative services like medication assisted treatments and support groups, or to harm reduction services like clean syringes, Narcan, and injection equipment.

### Long-term impacts of receiving poor healthcare in prison

Formerly incarcerated participants disclosed that they experienced health problems in prison that were inadequately addressed by medical staff, or in some cases were not addressed at all. Participants described the healthcare system in prison as being characterized by delayed care, exploitative costs, and lack of quality medical care, and stated that negligence and systemic issues gave rise to long-term negative health consequences. Participants also described feeling dehumanized as patients to the extent that nurses and physicians “don’t even want to touch [them]” to properly evaluate their medical conditions. Tanya stated that, in her experience, “the staff just treats everybody like they’re bullshitting when they actually need medical help… they don’t help people until they’re like on the floor like dying… or bleeding.” Kenneth described that for pain, fever, and other seemingly “non-emergent” situations, they “have to fill out a form and 24 hours later they might be called in depending on the severity [of their condition], and then wait for a couple of hours before seeing the doctor.” During that initial visit “if you don’t have the symptoms any longer, they write you up for submitting a false report.” Participants recalled being charged anywhere from $5-$12 copay per visit, which is financially burdensome given that people primarily rely on “somebody [sending them] money.” In Florida, the average pay for people who work while incarcerated is $0.20 to $0.55 per hour [35].

All formerly incarcerated participants shared that they had to serve as self-advocates for basic medical care during their time in prison. Aaliyah, a middle-aged woman, described herself as healthy prior to entering prison. However, while in prison, she was walking to breakfast at 5:30 AM. It was dark, and she describes the asphalt as having many potholes. As she walked to breakfast in the chunky clogs that prisoners wear, she tripped on a pothole and fell. Aaliyah said:

“I had to catch myself with my hands and bent my fingers–it bent my fingers–the impact. It was on a steep hill and the impact bent my fingers back and split one of them open. So, I got 5 stitches in my finger and then they didn’t do an X-ray or anything. Even though I’m bleeding and everything and they had to stitch me up. They didn’t do an X-ray, they didn’t see it was necessary, and sent me on my way, and that’s kind of how they do things there. They don’t, they don’t—they will only do what’s absolutely necessary. I wouldn’t even have seen the doctor if I hadn’t been bleeding, but you know they’re afraid of blood, so haha. Thank goodness I got stitches… But anyways, my hand folded up like a catcher’s mitt within a couple of days and um they still didn’t want to do anything. Didn’t want to see me. Um finally, the um the the colonel, I believe is the one that got me in there… I kept writing and saying, hey, my hand is huge, like I need an X-ray and they still wouldn’t see me. It took—it took I want to say at least a week before they brought me in for X-rays, and then they told me nothing was broken and they just gave me some ice twice a day for like three days and told me nothing was wrong. But meanwhile, I couldn’t—my fingers, I couldn’t bend them. They were swollen, and they were crooked. They look broken. In fact, to this day, my middle finger looks broken.”

Only after 2 months of “pushing and pushing and pushing” for treatment of her swollen and fractured hand did the assistant warden transfer Aaliyah to a medical facility for care. She felt that her broken finger had been brushed under the rug and improperly treated. Furthermore, Aaliyah speculated that the prison refused to take X-rays of her hand to avoid validating her concerns. In other words, if there was no X-ray, there was no broken finger. Aaliyah still struggles with her hand and accessing healthcare post-release. She continues:

“I think I have ligament damage as best I can tell from what I’ve just looked on the internet myself, but I haven’t been able um to afford probably till now to go to a doctor on my own and still I don’t have health insurance and um if I had to go to a specialist, there’s no way I can afford it and you know… um just, my hand is where it is.”

Kenneth, a man in his late 50s, had a similarly negative healthcare experience:

“When I, when I had a heart attack in there, the uh, the the the staff officer of the unit, um, didn’t believe I was having a heart attack, and so she didn’t call for medical response. So I had to wait until her shift was over. And then the lady that came on duty after her shift, then she um had to call the doctor because they don’t have blood pressure cuffs inside the unit. They have to come from medical—from a different location. So, they didn’t want to bring them—the medical cuffs—and have to deal with a heart attack at the same time. They were, they were kind of tossed up and so when they finally did bring the blood pressure cuffs, my blood pressure was, was way up. Was way up. And she had to recite it to them like several times. And they were like, that was too high. So, then they had to do another call for the heart attack to get to medical. And then I had to wait until I got to medical before they gave me nitroglycerin.”

When asked why Kenneth had to wait so long to receive medical attention for his heart attack, he responded:

“I think I had the heart attack at like 9 or 10 o’clock at night and then I had to wait for the next shift for 11 o’clock personnel to come on duty, and then when they came on duty, they had to call medical and they were tossed up as to whether to send a blood pressure cuff or just bring me in as a heart attack victim. So, it took like three hours from the time I initially had the heart attack… they don’t care. You’re, you’re no longer a person. You’re a body. That’s all.”

Kenneth went on to explain that another inmate was in a similar situation but was not so fortunate. A fellow inmate who had a heart attack passed away because he was not treated in time. Recounting the three-hour wait time he endured between the onset of his heart attack and the administration of nitroglycerin, Kenneth cited bureaucracy and the pervasive culture of disbelieving the legitimate medical needs of incarcerated individuals as a key problem in the correctional health system.

The lack of autonomy in being able to control their diet or medical care was described as dehumanizing, with participants expressing that the sense of dependency they experienced during incarceration felt “crippling”. When recounting her newly developed cardiovascular disease, Aaliyah stated that she was “put on Lipitor, which I really resisted. I did not want to take it, but couldn’t control my diet,” ultimately leading her to use medication to treat her condition rather than having the choice to modify her diet. Kenneth described the taxing mental health trauma associated with the lack of transparency in what medications they were being administered, citing feeling “unsafe from the staff” and expressing concern that he “can’t identify what [medications] they were giving him.” Moreover, as a diabetic, Kenneth needed regular access to nutritious foods, but was unable to keep food like oranges, apples, or bread in his locker to help mitigate his hypoglycemic episodes. He relayed that fruits are considered contraband beyond the scarce amounts provided in the dining hall, forcing patients like Kenneth to either place their health at risk, or risk facing additional punishment. Kenneth stated that “sometimes I was dizzy and I’d start swooning, and if I could eat an orange or apple or something like that, that would make me feel a little better and I wouldn’t fall out.” But, if Kenneth was caught with apples or oranges he would get in trouble because “you’re not supposed to take food, period. The only time they let you keep food was what you bought in the store, and they didn’t sell fresh fruit in the store. They only sell salt, supplements, like like soups, ramen soups with a lot of salt or cookies, pastries. Um sometimes meats, but the meats that they sell in the store are not really seasoned. They don’t have fruit.”

Overall, formerly incarcerated participants described their incarceration experiences as compromising their physical and mental health, causing them to develop chronic conditions that persist into reentry. Professionals like Jackson, a substance use treatment facilitator, have observed firsthand the adverse long-term effects of incarceration on reentrants, and assert that “[correctional institutions’] job is to keep [incarcerated individuals] there until their time is done and then let them go… they’re not necessarily concerned too much about helping these people.”

## Discussion

We identified several social and structural barriers and facilitators to health during reentry. Access to housing, employment, and food are three important social determinants of health and were identified as barriers to health during reentry. Affordable housing is a major problem in Miami and restrictions on low-income housing and shelters make it even more difficult for returning citizens to find safe and stable housing. Moreover, there are over 1,000 collateral consequences of a criminal conviction in Florida, including 20 related to housing, 76 related to education, and 745 related to employment [36]. For example, Florida statute allows for people convicted of offenses “detrimental to other residents” to be evicted from mobile park homes [37]. This makes access to employment difficult as well due to legal discrimination against people with a criminal conviction. Without a stable income, access to health and nutritious food is difficult. Participants identified empowerment and social support and addressing trauma as potential facilitators to positive health post-release. Participants described how learning more about the inequities of the criminal legal system and learning to no longer be ashamed of their “felon” status was empowering for them. Unaddressed trauma was identified as a major issue, either trauma experienced prior to or during incarceration, and receiving trauma-informed counseling better positioned people to deal with day-to-day challenges.

People leaving prison have high rates of chronic illness and other conditions. Within Miami, three challenges with accessing medical care following release from prison were identified. First, participants discussed difficulties with accessing their medical records from the DOC and their frustration with having to “start over” on known diagnoses. Related, Florida is one of 12 states that are not Medicaid expansion states. Therefore, access to health coverage is difficult especially for returning citizens. The healthcare safety nets that do exist in the community were described as so bureaucratic that most people “give up.” Participants also noted that there was a lack of coordinated continuity of care, which can be especially harmful for people living with HIV and other chronic illnesses. Finally, formerly incarcerated participants described how the healthcare that they received in prison was of such poor quality that they were concerned about negative long-term health impacts. This concern was compounded by their inability to access medical care in the community.

Based on accounts from both groups of participants, it is clear that a holistic approach to reentry that addresses the social determinants of health is necessary. We have several recommendations for improving services. First, developing reentry resource guides that span holistic continuums of care (e.g. behavioral health services, primary care, support groups, safe housing programs, and job training programs) can support both reentrants seeking essential services and professionals who would like to present reentrants with opportunities for case management and linkage to care [38, 39]. Having a publicly accessible database of resources [40] that offers quality care that is affordable and non-discriminatory towards justice involved individuals [41–43] can counteract barriers that exist in reentry, ultimately breaking the silos that exist in the current reentry service landscape.

Second, developing holistic reentry support programs that offer educational programming and reentry support services that begin in correctional institutions pre-release and continue throughout the reentry period can support the transition back to civil society. Supplementing pre-release educational programming with linkage to essential services [44] and community providers post-release can heighten service utilization during reentry. Third, expanding the eligibility criteria of essential governmental aid programs such as Medicaid [45, 46] and Section 8 HUD Housing [47–49] to include coverage for justice involved individuals can help them secure affordable healthcare and housing upon release. Twelve states in the US, including Florida, have yet to expand Medicaid eligibility to include low-income adults without dependent children [50]. States that have expanded Medicaid eligibility to this population have cited increases in outpatient care utilization for chronic and preventable diseases [52], increases in the use of preventative health screenings and services [51], increases in outpatient substance use disorder treatment for reentrants three months post release [52], and reduced financial strain for patients seeking care [51]. Wisconsin’s Department of corrections implemented prerelease Medicaid enrollment assistance, which increased Medicaid enrollment one month post release by 25% for those who completed applications in prison pre-release [53].

While this study was successful in examining the health and health related needs of reentrants from a diverse stakeholder population, there were numerous potential limitations. Our study inclusion criteria was limited to individuals who re-entered into Miami-Dade County, so while the results are valid in characterizing the obstacles experienced by returning citizens in the reentry period, they are not generalizable to the experiences of reentrants in other regions or states with different social and political contexts. Another potential limitation is that many reentrants were incarcerated and released during the COVID-19 pandemic, so their experiences both in prison and during reentry may reflect additional barriers that are more severe than those experienced by reentrants in existing literature from the pre-COVID-19 pandemic era. We did not collect data on the duration of incarceration for each of the formerly incarcerated respondents, and this may have impacted the health status and healthcare needs of respondents upon release, as more severe health outcomes may exist among those who have been incarcerated for a longer period. Among our study’s strengths, the most robust is that we included diverse perspectives among our respondent sample to achieve a more holistic understanding of the reentry period, with respondents encompassing formerly incarcerated individuals, judges, substance use support group leaders, harm reduction specialists, and reentry program leaders who serve diverse populations of returning citizens. We believe this research can better inform future reentry programs, reentry support services, and correctional health system reform to better address the health and health related needs of returning citizens within the South Florida region.

## Conclusion

This study provides a foundation for examining the healthcare needs of reentrants to Miami, and offers insight about the social and structural barriers that prevent them from fulfilling the social determinants of health post-release. By conducting in-depth interviews and examining the perspectives of both reentrants and professionals, we provide a holistic overview of the long-term consequences of incarceration on reentrant health. This information can be used to inform future attempts to support reentrants returning to Miami as they work to re-integrate and address their healthcare needs following incarceration. Based on participant data, it is clear that improving the health and wellbeing of justice impacted and other marginalized people in Miami can be achieved by addressing community health needs [54], established health inequities [55, 56] and long-standing structural inequalities [57–59]. Engaging in these public health efforts will have the added benefit of reducing reliance on the carceral system to address social problems [60].

## Supporting information

S1 Appendix Semi-structured interview guide for professional sample. (DOCX)

S2 Appendix Semi-structured interview guide for formerly incarcerated participants. (DOCX)
