# Supplementary material for: Barriers and facilitators to health during prison reentry to Miami, FL
Source: PLoS One. 2023 Oct 30;18(10):e0285411. doi: 10.1371/journal.pone.0285411 (PMC10615260; doi:10.1371/journal.pone.0285411)
Supplement: S1 Appendix — (DOCX) [file pone.0285411.s001.docx]

Semi-structured interview guide for professional sample

I would first like to ask you questions about **you**! First,

1. What do you do professionally?
2. Could you tell me more about your experiences with working with formerly or currently incarcerated individuals?
3. Could you describe the most important lessons you learned through working with this population?

Let’s move onto the **needs** in this community,

1. Given your expertise, what do formerly incarcerated individuals need the most? It does not have to be limited to healthcare.

Now I would like to ask you about the **barriers**:

1. What do you think are the greatest challenges that formerly incarcerated individuals face?
2. What are the barriers that you had when working with formerly incarcerated individuals?
3. How did you overcome those barriers?
4. Has any specific organization been helpful? What did ____ help you with?
5. What do you think is missing in Miami Dade county for this population?
6. What kind of services do you wish to see in our Transitions clinic?
7. Is there something else you think I should know to understand the formerly incarcerated individuals better?
8. After having these experiences, what advice do you have for us?
9. Is there something you might not have thought about before that occurred to you during this interview?

Thank you for taking your time to talk to us about your experience. Do you have any questions for me?
