# Supplementary material for: Barriers and facilitators to health during prison reentry to Miami, FL
Source: PLoS One. 2023 Oct 30;18(10):e0285411. doi: 10.1371/journal.pone.0285411 (PMC10615260; doi:10.1371/journal.pone.0285411)
Supplement: S2 Appendix — (DOCX) [file pone.0285411.s002.docx]

Semi-structured interview guide for formerly incarcerated participants

**What was your experience in prison like?**

**Probe questions:**

1. How much time did you serve in prison?
2. What prison(s) were you in?
3. What was your experience inside?
4. How was orientation?
   1. Were you allowed to go to the restroom?
5. How were you treated in prison?
6. What was your average day like?
7. How many meals was available?
   1. How was the food?
8. Did you have toothbrushes and toothpaste?
   1. Did you get a new toothbrush every six months/year?
9. What was work like in prison?
   1. Did you have any protection when you worked?
      1. ex. when you worked outside in the sun, did you have a hat or sunscreen?
10. Were you shipped around a lot?
11. Did you feel like you were supposed to be in prison?
12. What does it mean to be institutionalized?

**What does health mean to you?**

1. What does healthcare mean to you?
2. What does being healthy look like to you?
3. What does physical health mean to you?
4. What does mental health mean to you?
   1. Was there counseling when you were going through mental issues?
   2. How easy was it to access psychological help?
   3. Were you ever in solitary confinement?
   4. Did you lose anyone close to you when you were in prison? (family that passed away?)
5. How did you say healthy in prison?
   1. Were you allowed to run in prison?
   2. Did you exercise?

**GENDER**

- **Men**

1. Did you feel safe in prison?
2. Did you feel respected in prison?
3. Was there anything that men need in prison?
4. Did you have toilet paper?

- **Women**

1. Did you feel safe in prison?
2. Did you feel respected in prison?
3. What are some things that you think women need in prison?
4. How was it getting your period in prison?
5. Was it easy for you to access products when you had your period? Like tampons or pads?
   1. needed to ask for pads, no tampons, no good pads. Only a certain amount of pads.
   2. How many pads did you get?
   3. Did you feel degraded as a women when you had to ask?
6. Did you have toilet paper?
   1. How much toilet paper could you use?

**Getting a sense of their general health care needs**

1. Can you tell me a little bit about yourself and your health?
   1. Do you have any pre-existing medical issues?
   2. Any other health issues?
   3. When did it start?
2. Any other health problems that you have?
3. How do you take care of your health?

**Were your healthcare needs being met in prison?**

1. How was the medical facility in the prison?
2. Did you get the treatment you needed in prison?
3. If you had a problem with health stuff, how did you go about getting treatment? Can you walk me through what you would do to get treatment/meds/ or help?
4. How was it when you put a request?
   1. How fast/slow were your requests met?
5. Did you have to pay for the medical visit?
   1. Did they take your prison money for the medical visit?
6. How did you deal with mental health in prison?
   1. Did you ever want to commit suicide in prison?

**What the transition to community health care was like?**

1. How has it been being out of prison?
2. How has your health been since you left prison?
3. If you have a problem with health stuff, how do you get treated now? Can you walk me through this?
4. How has your experience been with clinics and health care providers outside of prison?
   1. Can you get meds?
   2. Have you been able to see a doctor when you need to?
   3. Did anyone follow up with you?
5. Do you have any health insurance?
6. How was it finding someone who accepts your insurance?

**Social services used more generally upon release?**

1. How has life been since you’ve been out?
2. How do you get what you need to live and be healthy?
3. Do you use social services like clinics, food banks, or shelters?
4. How has your experience been with these organizations?
5. Did you have a support group when you got out?
   1. who/what was your support group?
   2. How did they help you?

**What has been helpful or unhelpful to you?**

1. What has been the hardest part of accessing health clinics, medications, treatments, etc.?
2. What organizations have helped you the most?
3. I want you to reflect for a second. What does good healthcare look like for you?

**Transition to society?**

1. What did you leave prison with?
2. How have you adjusted since being released?
3. Would you say youve got back to normal life?
4. How has probation been like?
5. How easy was it to access drugs when you got out?
6. How easy was it to access healthcare when you got out?
7. How easy was it to access a job or housing when you got out?
8. How did you feel being called by your name after being called an inmate/ a number for so long?
9. Did you have any referrals once you were out?
10. How did you transition to being free? Do you feel mentally free?
    1. How did you restore your rights?
